# Shrimp miR-12 Suppresses White Spot Syndrome Virus Infection by Synchronously Triggering Antiviral Phagocytosis and Apoptosis Pathways

**DOI:** 10.3389/fimmu.2017.00855

**Published:** 2017-07-31

**Authors:** Le Shu, Xiaobo Zhang

**Affiliations:** ^1^College of Life Sciences, Laboratory for Marine Biology and Biotechnology of Qingdao National Laboratory for Marine Science and Technology, Zhejiang University, Hangzhou, China

**Keywords:** microRNA, antiviral immunity, phagocytosis, apoptosis, degradation of target microRNA

## Abstract

Growing evidence has indicated that the innate immune system can be regulated by microRNAs (miRNAs). However, the mechanism underlying miRNA-mediated simultaneous activation of multiple immune pathways remains unknown. To address this issue, the role of host miR-12 in shrimp (*Marsupenaeus japonicus*) antiviral immune responses was characterized in the present study. The results indicated that miR-12 participated in virus infection, host phagocytosis, and apoptosis in defense against white spot syndrome virus invasion. miR-12 could simultaneously trigger phagocytosis, apoptosis, and antiviral immunity through the synchronous downregulation of the expression of shrimp genes [PTEN (phosphatase and tensin homolog) and BI-1(transmembrane BAX inhibitor motif containing 6)] and the viral gene (*wsv024*). Further analysis showed that miR-12 could synchronously mediate the 5′–3′ exonucleolytic degradation of its target mRNAs, and this degradation terminated in the vicinity of the 3′ untranslated region sequence complementary to the seed sequence of miR-12. Therefore, the present study showed novel aspects of the miRNA-mediated simultaneous regulation of multiple immune pathways.

## Introduction

During virus infection, multiple immune pathways are employed by hosts to defend against virus invasion (1). As the first line of the immune systems, innate immunity plays an essential role in immune responses against virus infection in which phagocytosis and apoptosis are two important components (1). Studies have shown that phagocytosis is required for host antiviral immunity through the direct rapid engulfment of virions and apoptotic cells (2–4). Phagocytosis can be divided into multiple stages, including recognition between particles and cell receptors, actin polymerization to engulf exogenous substrates and transfer of a newly formed phagosome into a mature phagolysosome through the by removal of the actin coat (5). In invertebrates, Rab6 protein, which directly interacts with actin, is essential for the correct conformation of actin, thus showing a critical effect on phagocytosis against virus infection (4). During virus infection, a series of signaling pathways can be triggered in the host, leading to apoptosis of infected cells (6, 7). The apoptotic cells attract specialized phagocytes and are subsequently devoured by phagocytes (8, 9). Avian influenza virus encodes NS1, a viral protein with a four-amino acid sequence at its carboxyl terminus termed the PDZ-binding motif. Recent reports have shown that this PDZ-binding motif can protect virus-infected cells from apoptosis by directly disrupting the proapoptotic function of scribble, thereby inhibiting infected cell elimination by immune cells (10). To protect the host from virus invasion, animals have developed various signaling pathways. However, whether an individual molecule can synchronously trigger various antiviral pathways remains unknown. As key regulatory elements of gene expression, an individual microRNA (miRNA) possess multiple target genes involved in different signaling pathways (11, 12), implying that a miRNA may simultaneously trigger multiple antiviral pathways.

MicroRNAs, typically ~22 nucleotides in length, are members of an extensive type of small non-coding RNAs, which exert physiological functions through direct binding to the 3′ untranslated regions (3′UTR) of mRNAs, resulting in mRNA degradation or destabilization (13). In splenic macrophages under hypersplenism, miR-615-3p is highly expressed and targets the ligand-dependent nuclear receptor corepressor (LCoR), an inhibitor of phagocytosis-promoting protein peroxisome proliferator-activated receptor gamma (PPARγ), thus upregulating PPARγ and eventually enhancing the phagocytic capacity of splenic macrophages (14). For the regulation of apoptosis, miR-101 is downregulated in human hepatocellular carcinoma and can markedly inhibit the expression of the antiapoptotic factor Mcl-1 (BCL2 family apoptosis regulator), leading to apoptosis of hepatocellular carcinoma cells (15). Currently, the miRNA-mediated regulation of apoptosis and phagocytosis represent important mechanisms in the host to defend against virus invasion. In shrimp, the host miR-7 directly targets the viral early gene *wsv477* of white spot syndrome virus (WSSV) leading to the repression of WSSV infection (16). Previous studies have reported that WSSV infection leads to the accumulation of miR-965, which diminishes the ATG5 expression, thus enhancing host antiviral phagocytosis (12). Currently, miRNA-mediated gene expression regulation is generally a one-to-one type of regulation (i.e., one miRNA to one mRNA). However, the miRNA-mediated regulation mechanism of multiple target gene expressions has not been extensively investigated.

To elucidate whether a single miRNA can simultaneously trigger multiple antiviral pathways by targeting different genes, shrimp miR-12, which has been implicated in antiviral phagocytosis and apoptosis in shrimp (17), was characterized in the present study. In recent years, WSSV has attracted increasing attention as a model for virus–host interactions *in vivo* (18). In the present study, the results indicated that miR-12 could simultaneously trigger antiviral phagocytosis and apoptosis and inhibit the virus proliferation by synchronously targeting the host genes *PTEN* and *BI-1* and the viral gene *wsv024*.

## Materials and Methods

### Shrimp Culture and Virus Infection

Shrimp *Marsupenaeus japonicus* (10–12 cm in length) were cultured at 20°C in tanks with seawater. For each treatment, 20 randomly selected individuals were raised in an 80 l aquarium. To ensure that the shrimp were virus-free prior to experiments, the shrimp hemocytes were subjected to PCR detection using WSSV-specific primers (5′-TATTGTCTCTCCTGACGTAC-3′ and 5′-CACATTCTTCACGAGTCTAC-3′). The virus-free shrimp were intramuscularly injected with 0.1 ml of WSSV solution (10^5^ copies/ml) using a syringe with a 29-gauge needle. The WSSV solution was obtained from WSSV-infected shrimp (18). At various times postinfection, the shrimp hemocytes were collected for further analysis.

### Northern Blot Analysis

Total miRNAs were extracted from shrimp hemocytes using a mirVana miRNA isolation kit (Ambion, USA) following the manufacturer’s instructions. The RNAs were separated on a denaturing 15% polyacrylamide gel containing 7 M urea in 1× TBE buffer (90 mM Tris–boric, 2 mM EDTA, pH 8.0) and subsequently transferred to a Hybond-N+ nylon membrane. After UV cross-linking, the membrane was prehybridized in DIG (digoxigenin) Easy Hyb granule buffer (Roche, Basel, Switzerland) for 0.5 h at 42°C and then hybridized with DIG-labeled miR-12 (5′-ACCAGTACCTGATGTAATACTCA-3′) or U6 (5′-GGGCCATGCTAATCTTC TCTGTATCGTT-3′) probe at 42°C overnight. The DIG labeling and detection were performed using the DIG High Prime DNA Labeling and Detection Starter Kit II (Roche, Germany) according to the manufacturer’s instructions.

### Silencing or Overexpression of miR-12 in Shrimp

To overexpress or knock down the expression of miR-12 in shrimp, miR-12 (5′-T GAGTATTACATCAGGTACTGGT-3′) synthesized using the *in vitro* transcription T7 kit (TaKaRa, Japan) or the synthesized anti-miR-12 oligonucleotide (AMO-miR-12) (5′-ACCAGTACCTGATGTAATACTTCA-3′) was injected into shrimp at 30 μg/shrimp. As controls, the sequence of miR-12 or AMO-miR-12 was randomly scrambled, generating miR-12-scrambled (5′-ACTCATAATGTAGTCCAT GACCA-3′) or AMO-miR-12-scrambled (5′-TGGTCATGGACTACATTATGAGT-3′). All synthesized miRNAs were dissolved in miRNA solution (50 mM Tris–HCl, 100 mM NaCl, pH 7.5) and quantified using NanoDrop ND-100 spectrophotometer. At different times after injection, three shrimp were randomly selected from each treatment, and the selected shrimp hemocytes were collected and mixed for later use. The above assays were biologically repeated three times.

### Analysis of WSSV Copies Using Quantitative Real-time PCR

To evaluate the proliferation of WSSV in shrimp, the quantitative real-time PCR was performed using WSSV-specific primers (5′-TTGGTTTCATGCCCGAGATT-3′ and 5′-CCTTGGTCAGCCCCTTGA-3′) and a TaqMan fluorogenic probe (5′-FAM-TGCTGCCGTCTCCAATAMRA-3′). The PCR mixture (10 µl) consisted of 5 µl Premix Ex Taq (TaKaRa, Japan), 200 ng DNA template, 0.2 µl of 10 µM primers, and 0.2 µl of 10 µM TaqMan fluorogenic probe at a final concentration of 0.2 µM. The DNA template was extracted from the shrimp hemocytes using an SQ tissue DNA isolation kit (Omega Bio-tek, Norcross, GA, USA) according to the manufacturer’s instructions. A plasmid containing a 1,400-bp DNA fragment from the WSSV genome was used as the reference plasmid as previously described (19). The PCR conditions were 95°C for 1 min, followed by 45 cycles at 95°C for 30 s, 52°C for 30 s, and 72°C for 30 s.

### Phagocytosis Assay with Fluorescein Isothiocyanate (FITC)-Labeled WSSV

To label WSSV virions with FITC, the purified virions were incubated in 1 mg/ml FITC (Sigma, USA) solution (dissolved in 0.1 M NaHCO_3_, pH 9.0) for 1 h at room temperature, followed by washing with 0.1 M NaHCO_3_. The shrimp hemocytes were rinsed with cold PBS (50 mM Tris–HCl, 100 mM NaCl, pH 7.5). To perform the phagocytosis assay, the FITC-labeled WSSV virions were mixed with shrimp hemocytes at a ratio of 50:1 (WSSV copies: cell numbers) and subsequently incubated at 28°C for 30 min. The hemocytes were rinsed with PBS to remove unphagocytosed FITC-labeled WSSV virions. After centrifugation at 200 × *g* for 10 min, the hemocytes were resuspended in 1% paraformaldehyde (Sigma, USA). Finally, the hemocytes were analyzed using flow cytometry (Beckman Coulter, USA). For each sample, 10,000–20,000 hemocytes were assessed. The experiments were biologically repeated three times.

### Annexin V Analysis

The detection of shrimp hemocyte apoptosis using annexin V (Invitrogen, USA) was conducted following the manufacturer’s instructions. Shrimp hemocytes were washed with cold PBS. Then the hemocytes were incubated in 1× annexin binding buffer at 1 × 10^6^ cells/ml, followed by the addition of 5 µl of Alexa Fluor 488-annexin V and 1 µl of 100 µg/ml propidium iodide (PI) in the dark. After incubation for 15 min, 400 µl of 1× annexin binding buffer was added to each sample to stop the reaction. The sample was analyzed using flow cytometry.

### TUNEL (Terminal Deoxynucleotidyl Transferase-Mediated dUTP-Biotin Nick End Labeling) Assay

Shrimp hemocytes were collected and separated on poly-l-lysine-coated glass slides (Sigma), followed by standing for 10 min at 4°C. The hemocytes were fixed in 4% paraformaldehyde for 25 min at 4°C. The fixed hemocytes were washed with cold PBS and then subjected to the permeabilization with 0.2% Triton X-100 for 5 min. Next, the hemocytes were equilibrated in 100 µl of equilibration buffer at 4°C for 10 min. The equilibrated hemocytes were counterstained with PI after incubation with rTdT mix in a humid environment for 1 h. Subsequently, 2× SSC (1× SSC is 0.15 M NaCl and 0.015 M sodium citrate) was added to the slide to stop the reaction. The slide was covered with antifade solution (Invitrogen) to prevent signal quenching.

### Evaluation of Caspase 3/7 Activity

The apoptosis of shrimp hemocytes was detected using a Caspase-Glo3/7 assay (Promega, USA) according to the manufacturer’s instructions. Briefly, shrimp hemocytes were harvested and washed with PBS. Subsequently the hemocytes were incubated with Caspase-Glo 3/7 reagent at room temperature for 2 h. The caspase 3/7 activity was measured using a Synergy 2 Multi-Mode microplate reader (BioTek).

### Prediction of Genes Targeted by miR-12

To predict the target genes of miR-12, four algorithms including TargetScan, miRanda, Pictar, and miRInspector were used to predict the targeted sites in the 3′UTRs of the WSSV genes and the shrimp genes as previously described (20).

### Plasmid Construction

To explore the direct interaction between the predicted targets and miR-12, the 3′UTR of a predicted target gene was cloned into the pIZ/V5-His vector (Invitrogen, USA), generating a recombinant plasmid containing EGFP and the 3′UTR of a predicted target. As a control, the 3′UTR sequence matching the seed sequence of miR-12 was scrambled to produce the mutant construct. All the recombinant plasmids were confirmed through sequencing.

### Cell Culture, Transfection, and Fluorescence Assays

Insect High Five cells (Invitrogen) were cultured in Express Five serum-free medium (Invitrogen) containing l-glutamine (Invitrogen) at 27°C. At 70% confluence, the cells were co-transfected with 2 µg of EGFP, EGFP-target gene 3′UTR or EGFP-Δtarget gene 3′UTR and 100 pM of miR-12 or miR-12-scrambled using Cellfectin transfection reagent (Invitrogen) according to the manufacturer’s instructions. The miRNAs were synthesized at Shanghai GenePharma Co., Ltd. (Shanghai, China). At 48 h after co-transfection, the fluorescence intensity of cells was assessed using a Flex Station II microplate reader (Molecular Devices, USA) at 490/510 nm excitation/emission (Ex/Em). The experiments were biologically repeated three times.

### Quantification of mRNA Levels with Real-time PCR

The mRNA levels of *wsv024, PTEN, PARP*, and *BI-1* were quantified with real-time PCR using sequence-specific primers (wsv024, 5′-CATCCTGTTAGAGT TTCCTGTTTC-3′ and 5′-ATATTACTGCCATGTTATCTGTTGC-3′; PTEN, 5′-CCAAAATAACCACAACAACAC-3′ and 5′-CACTTCCTGCTCTCCCTT-3′; PARP1, 5′-GAAGAATTACAACTGCGTCCTG-3′ and 5′-GTTCCTCGAAATGGG CTATG-3′; BI-1, 5′-TGCGGCTTCATTGTTTACG-3′ and 5′-CACGGTTCTTCCTCTTGTTCTG-3′; and β-actin, 5′-CGAGCACGGCATCGTTACTA-3′ and 5′-TTGTAG AAAGTGTGATGCCAGATCT-3′). Shrimp hemocytes were collected and subjected to total RNA extraction using the RNAprep Pure Cell/Bacteria kit (Tiangen Biotech Co. Ltd., Beijing, China). The cDNA was synthesized using the PrimeScript™ 1st strand cDNA synthesis kit (Takara, Japan). Quantitative real-time PCR was performed in a total volume of 25 µl containing 5 µl of SYBR^®^ Premix Ex Taq, 0.5 µl of 10 µM forward and reverse primers, and 100 ng of cDNA template. The PCR conditions were 95°C for 1 min, followed by 40 cycles at 95°C for 15 s and 60°C for 45 s.

### RNAi Assay *In Vivo*

According to the gene sequence, siRNAs specifically targeting these genes were synthesized, generating the corresponding siRNAs (wsv024-siRNA, 5′-CGAUGAG UACUUGUCUAGCGUUUAA-3′; PTEN-siRNA, 5′-UAGAGUAGCAGAUGUU UGAAGUGUA-3′; and I-1-siRNA, 5′-GCAAACUGGAGAAAGUGCUUUCUGA-3′). As controls, the siRNA sequences were randomly scrambled, producing the corresponding scrambled siRNAs (wsv024-siRNA-scrambled, 5′-CGAAGUACUUG UCUAGCGUUUGUAA-3′; PTEN-siRNA-scrambled, 5′-UAGGAUGACGUAUUU AAGUGAGGUA-3′; BI-siRNA-scrambled, 5′-GCAGGUCAAGAGUGAUUUCCA AUGA-3′). All siRNAs were synthesized using an *in vitro* transcription T7 kit (TaKaRa, Japan) according to the manufacturer’s instructions. The synthesized siRNAs were quantified using spectrophotometry and dissolved in siRNA buffer (50 mM Tris–HCl, pH 7.5, 100 mM NaCl).

The shrimp were injected with siRNA (4 nM) or siRNA-scrambled (4 nM) and WSSV (10^4^ copies/shrimp). At different times after injection, the hemocytes of three randomly selected shrimp from each treatment were collected for later use.

### Western Blotting

The proteins were analyzed in a 12% SDS-PAGE gel and then transferred onto a nitrocellulose membrane (Bio-Rad, USA). The membrane was blocked in blocking buffer [3% bovine serum albumin in TBST (250 mM NaCl, 10 mM Tris–HCl, 0.5 ml/l Tween 20, pH 7.4)] for 2 h at 4°C. Subsequently the membrane was incubated with a primary antibody (anti-wsv024 IgG, anti-BI-1 IgG, anti-PTEN IgG, or anti-actin IgG) for 2 h at 4°C. After two washes with TBST, the membrane was incubated with HRP (horseradish peroxidase)-conjugated anti-mouse IgG (Sigma, USA) for 1 h at 4°C, followed by chemiluminescence detection with ECL substrate (Thermo Scientific, USA). All of the primary antibodies were prepared in our laboratory.

### miR-12-Mediated Degradation of Target mRNAs

The 3′UTRs of *wsv024, PTEN*, and *BI-1* mRNAs were amplified with specific primers (wsv024, 5′-TAATACGACTCACTATAGGTCTTCGTTAAAATCAGTTTTACCT-3′ and 5′-GTGCTAATAAACCAACGTCTTTC-3′; PTEN, 5′-TAATACGACTCACTATAGGGCGCACGAAGCTTTGGC-3′ and 5′-TCCTGTACACTACAAATAATAAGCCTC-3′; BI-1-F, 5′-TAATACGACTCACTATAGGGATGCCACA GATGTAATTTAGAAG-3′ and 5′-AATAGGATAATCACATGAAATTCTG-3′), which contained T7 promoter sequence at their 5′-ends of the forward primers, using a T7 transcription kit (TaKaRa, Japan) according to the manufacturer’s instructions. To get the Ago1 complex of shrimp, shrimp muscles were homogenized in lysis buffer [20 mM Tris–HCl, 150 mM NaCl, 1.5 mM MgCl_2_, 0.25% NP-40 (Non-idet P-40), and 1 mM PMSF (phenylmethanesulfonyl fluoride), pH 7.5] for 15 min on ice and subsequently centrifuged at 10,000 × *g* for 10 min at 4°C. The cell supernatants were incubated with the shrimp Ago1-specific antibody, prepared in our laboratory, for 1 h at 4°C, followed by mixing with protein-G-coupled agarose beads for 4 h at 4°C. After washes with cold PBS, the beads were resuspended in reaction buffer [100 mM KOAc, 40 mM 4-(2-hydroxyethyl)-1-piperazineethanesulfonic acid (HEPES), 5 mM MgCl_2_, 2 mM Dithiothreitol, 0.35% Triton X-100, and 1 mM PMSF, pH 7.6]. To investigate the miRNA-mediated degradation of target mRNAs, the co-immunoprecipitated products of shrimp Ago1, 3′UTR (*wsv024, PTEN*, and/or *BI-1*) (100 ng), and miR-12 were mixed at different concentrations and supplemented with 2 µl of 10 mM ATP and 2 µl of 2 mM GTP at a total volume of 20 µl. After incubation at 30°C for different times, the RNA samples were separated using 1% agarose gel electrophoresis and then transferred to a nylon membrane (GE Healthcare, USA). The RNAs were detected using a 5′ DIG (digoxigenin)-labeled probe (*wsv024*, 5′-TCACGGTTATGGCCAGCGCTACGC-3′; *PTEN*, 5′-GAACACAGAGCAGAG GGGATGATAG-3′; and BI-1, 5′-TCTCAAACTCATCTATATCATTC-3′).

### Sequencing of the Degraded mRNA 3′UTR Fragments

The mRNA 3′UTR of the miR-12 target gene was incubated with miR-12 (20 nM) and Ago1 complex at 30°C for 2 h. Subsequently, the degraded fragments were separated using 1% agarose electrophoresis. After recovery from the agarose gel, the RNAs were reverse transcribed into cDNAs using the PrimeScript™ II 1st strand cDNA synthesis kit with random 6 primer (TaKaRa, Japan). Then, the single-stranded cDNAs were transformed into double-stranded cDNAs using the second strand cDNA synthesis kit (Beyotime Biotechnology, China). The cDNAs were cloned into the pEASY^®^-Blunt simple cloning vector (Transgen Biotech, China) and subjected to sequencing.

### Statistical Analysis

All numerical data presented in the present study were analyzed using one-way analysis of variance to calculate the means and SDs of triplicate assays.

## Results

### The Antiviral Activity of miR-12 in Shrimp

**Figure 1:**
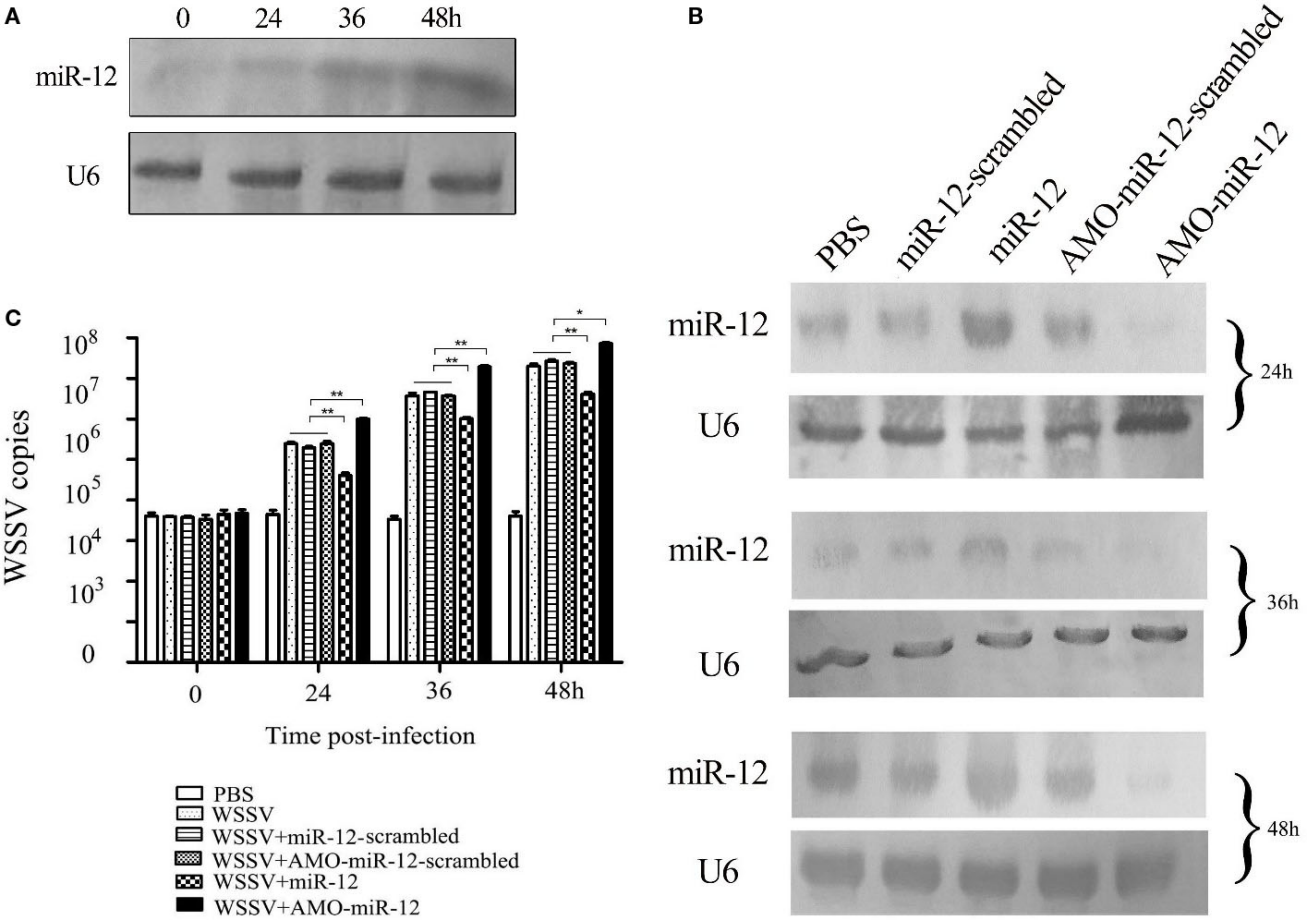
The antiviral activity of miR-12 in shrimp. **(A)** The expression profile of miR-12 in shrimp in response to virus infection. The shrimp were infected with white spot syndrome virus (WSSV). At different times postinfection, shrimp hemocytes were subjected to northern blot analysis to detect miR-12. U6 was used as a control. The numbers represent the time points postinfection. **(B)** The overexpression or silencing of miR-12 in shrimp. The virus-free shrimp were injected with miR-12 or AMO-miR-12, followed by detection of miR-12 expression using northern blot analysis. As controls, miR-12-scrambled and AMO-miR-12-scrambled were also injected. The probes are indicated on the left. U6 was used as a control. The numbers indicate the times after treatments. **(C)** The influence of miR-12 overexpression or silencing on virus infection. The WSSV copies of miR-12-overexpressed or miR-12-silenced shrimp were evaluated at various times postinfection using quantitative real-time PCR. The treatments are indicated on the right. In all panels, the statistical significance between treatments is indicated with asterisks (*p < 0.05; **p < 0.01).

To explore the role of host miR-12 in virus–host interactions, the expression level of miR-12 in WSSV-challenged shrimp was characterized. Northern blot analysis indicated that miR-12 was significantly upregulated in shrimp in response to WSSV infection, suggesting that miR-12 played important roles in host–virus interactions (Figure 1A).

To assess the roles of miR-12 in virus–host interactions, miR-12 expression was overexpressed or knocked down in shrimp, followed by the evaluation of virus infection. Northern blot analysis indicated that miR-12 expression in shrimp hemocytes was significantly upregulated from 24 to 48 h after the miR-12 injection compared with the controls (Figure 1B). The results showed that miR-12 overexpression led to significant decreases in WSSV copies in shrimp compared with the controls (WSSV, WSSV + miR-12-scrambled and WSSV + AMO-miR-12-scrambled) (Figure 1C). However, when miR-12 expression was inhibited by AMO-miR-12 (Figure 1B), the WSSV copies were dramatically increased compared with the controls (Figure 1C), indicating that miR-12 played a negative role in the virus infection. The results revealed that the miR-12 expression level in PBS-treated shrimp was identical to those of miR-12-scrambled and AMO-miR-12-scrambled-treated shrimp (Figure 1B), showing that there was no miR-12 or AMO-miR-12 toxicity in shrimp.

The above data implied that miR-12 possesses antiviral activity in shrimp.

### The miR-12-Mediated Suppression of WSSV Infection in Shrimp by Targeting the Viral *wsv024* Gene

To reveal the mechanism of miR-12-mediated antiviral immunity in shrimp, the WSSV genes targeted by miR-12 were characterized. The prediction results obtained using TargetScan, miRanda, Pictar and miRInspector algorithms showed that *wsv024* was a potential target of miR-12 (Figure S1A in Supplementary Material).

**Figure 2:**
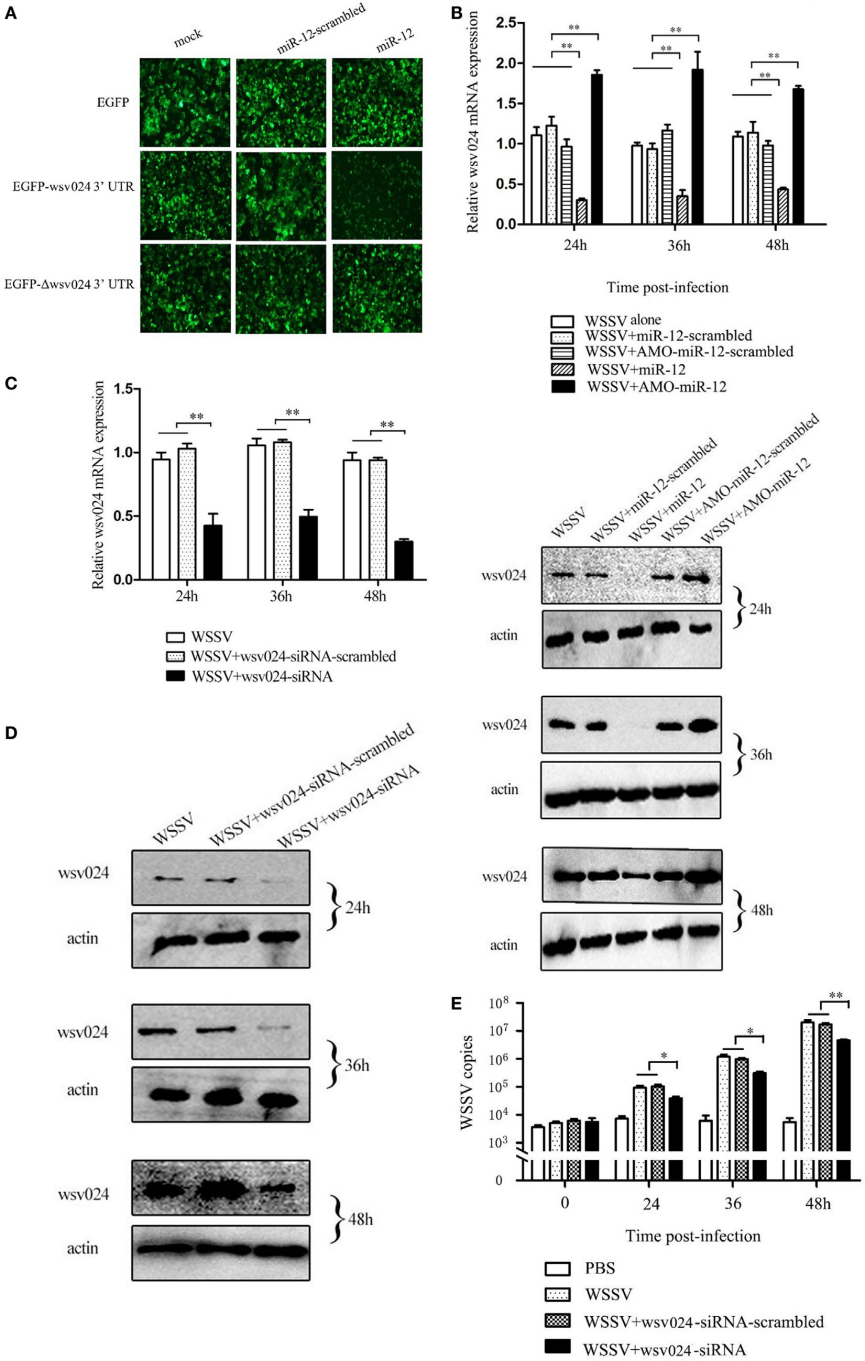
The miR-12-mediated suppression of white spot syndrome virus (WSSV) infection in shrimp by targeting viral *wsv024* gene. **(A)** The direct interaction between miR-12 and *wsv024*. Insect High Five cells were co-transfected with miR-12 or miR-12-mimic-scrambled with the recombinant plasmids, including EGFP-wsv024-3′UTR, EGFP-Δwsv024-3′UTR, and EGFP. At 48 h after co-transfection, fluorescent images were obtained. **(B)** The effect of the silencing or overexpression of miR-12 on *wsv024* expression in WSSV-infected shrimp. The shrimp were injected with miR-12 and WSSV, AMO-miR-12 and WSSV or WSSV alone. As controls, miR-12-scrambled and AMO-miR-12-scrambled were also injected. At different times after injection, shrimp hemocytes were subjected to quantitative real-time PCR (up) and western blotting (down) to evaluate the *wsv024* expression level. The numbers indicated the times postinfection. Shrimp β-actin was used as a control. **(C)** The knockdown of *wsv024* gene expression in shrimp. Shrimp were co-injected with WSSV and wsv024-siRNA. As controls, co-injections of WSSV + wsv024-siRNA-scrambled or WSSV alone were included in the assays. At different times postinfection, the *wsv024* mRNA level was determined using quantitative real-time PCR. **(D)** Western blot analysis of wsv024 silencing in shrimp. The numbers indicate the time points postinfection. **(E)** The effects of wsv024 silencing on virus infection. At different times postinfection, the WSSV copies in the wsv024-siRNA-treated shrimp were examined using quantitative real-time PCR. The statistically significant differences between treatments are represented with asterisks (*p < 0.05; **p < 0.01).

To assess the interaction between miR-12 and *wsv024*, a recombinant plasmid containing EGFP and wsv024 3′UTR (pIZ/EGFP-wsv024-3′UTR) was co-transfected with miR-12 into insect High Five cells (Figure S1B in Supplementary Material). The results indicated that the fluorescence intensity of the insect cells co-transfected with miR-12 and pIZ/EGFP-wsv024-3′UTR was remarkably decreased compared with the controls (Figure 2A), showing that miR-12 could directly target the *wsv024* gene.

To evaluate the interaction between miR-12 and wsv024 *in vivo*, miR-12 expression was upregulated or knocked down in WSSV-infected shrimp, followed by the detection of wsv024 expression levels. The results indicated the miR-12 overexpression decreased *wsv024* transcript levels in contrast with the controls (WSSV + miR-12-scrambled and WSSV alone) (Figure 2B). When the expression of endogenous miR-12 was reduced by AMO-miR-12, the *wsv024* gene expression was significantly upregulated compared with the controls (WSSV + AMO-miR-12-scrambled and WSSV alone) (Figure 2B). These data showed that miR-12 interacts with *wsv024 in vivo*.

To evaluate the influence of the interaction between miR-12 and wsv024 on virus infection, the expression of wsv024 was knocked down using wsv024-specific siRNA (wsv024-siRNA) in shrimp, followed by an assessment of WSSV proliferation. The results indicated that the expression of wsv024 was efficiently silenced by wsv024-siRNA compared with the controls (Figures 2C,D). In addition, wsv024 silencing resulted in remarkable inhibition of WSSV proliferation in shrimp (Figure 2E), indicating that wsv024 plays a positive role in WSSV infection.

Taken together, these findings showed that miR-12 functioned in the antiviral immunity of shrimp by targeting the WSSV *wsv024* gene.

### The Promotion of Shrimp Antiviral Phagocytosis Mediated by miR-12 *via* Targeting the Shrimp *PTEN* Gene

In a previous study, we revealed that shrimp miR-12 was implicated in antiviral phagocytosis and antiviral apoptosis in shrimp (17). To investigate the mechanism of antiviral phagocytosis and antiviral apoptosis mediated through miR-12, the shrimp genes targeted by miR-12 were predicted. As predicted, *PARP1* [*poly (ADP-ribose) polymerase 1*], *PTEN* (*phosphatase and tensin homolog*), and *BI-1* (*transmembrane BAX inhibitor motif containing 6*) genes were potential targets of miR-12 (Figure S2A in Supplementary Material). BI-1 and PARP1 are inhibitors of apoptosis (21, 22), while PTEN is a negative regulator of phagocytosis (23). In this context, the role of miR-12 in the antiviral phagocytosis of shrimp was characterized.

**Figure 3:**
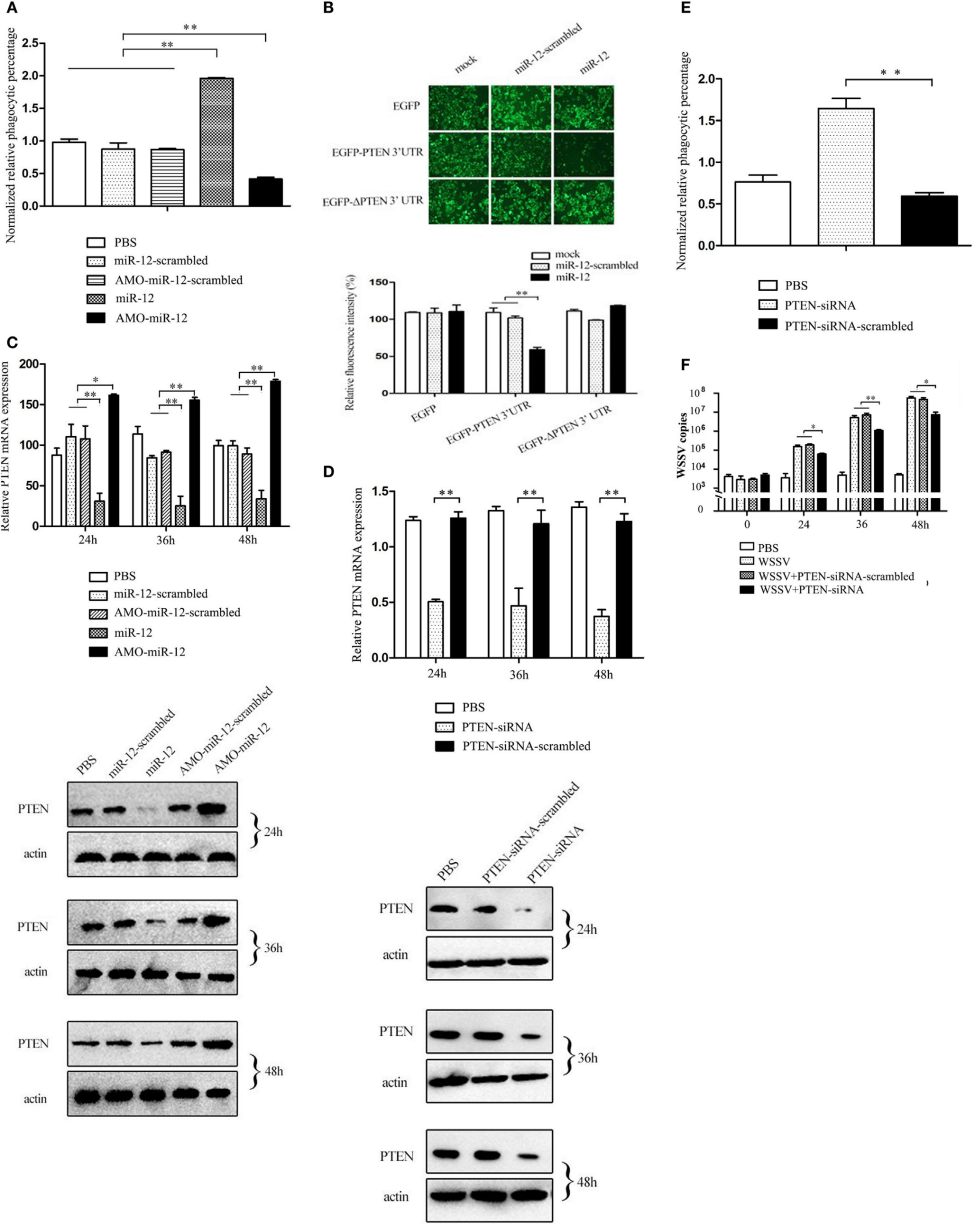
The promotion of shrimp antiviral phagocytosis mediated by miR-12 *via* targeting the shrimp *PTEN* gene. **(A)** The role of miR-12 in phagocytosis. At 36 h after injection, the phagocytic activity of shrimp hemocytes was assessed. The treatments are indicated on the right. **(B)** The direct interaction between miR-12 and PTEN. The insect cells were co-transfected with miR-12 and various constructs [EGFP, EGFP-PTEN 3′ untranslated region (3′UTR), or EGFP-ΔPTEN 3′UTR]. As a control, miR-12-scrambled was included in the transfection. At 36 h after transfection, the fluorescence intensities of the cells were examined. **(C)** The interaction between miR-12 and PTEN *in vivo*. Shrimp were injected with miR-12 or AMO-miR-12 to overexpress or silence miR-12. At different times after injection, the transcript level and protein level of *PTEN* in shrimp hemocytes were determined using quantitative real-time PCR (up) and Western blotting (down), respectively. In Western blots, the numbers represent the times postinfection. Shrimp β-actin was used as a control. **(D)** Knockdown of *PTEN* in shrimp. Shrimp were injected with PTEN-siRNA, followed by an assessment of PTEN expression using quantitative real-time PCR (up) and western blotting (down). PTEN-siRNA-scrambled was used as a control. **(E)** The role of PTEN in the antiviral phagocytosis of shrimp. Shrimp were injected with PTEN-siRNA or PTEN-siRNA-scrambled. At 36 h post-transfection, the phagocytic activity of shrimp against white spot syndrome virus (WSSV) was examined. **(F)** The effects of PTEN silencing on WSSV infection. The WSSV copy number in the PTEN-silenced shrimp was evaluated using quantitative real-time PCR at various times postinfection. In all panels, the data are presented as the means ± SD of three independent experiments. The significant differences between treatments are indicated with asterisks (*p < 0.05; **p < 0.01).

As shown in Figure 3A, when miR-12 was overexpressed, the phagocytic activity of shrimp hemocytes was significantly enhanced. However, miR-12 silencing remarkably decreased shrimp hemocyte phagocytic activity (Figure 3A). These results revealed that miR-12 exerted a positive effect on the antiviral phagocytosis of shrimp to fight against virus infection.

To reveal the mechanism of miR-12-mediated antiviral phagocytosis in shrimp, the interaction between miR-12 and its potential target *PTEN* gene was explored. Recombinant plasmids containing EGFP and *PTEN* 3′UTR or Δ*PTEN* 3′UTR were co-transfected with miR-12 or miR-12-scrambled into insect cells (Figure S2B in Supplementary Material). The results indicated that the fluorescence intensity of cells co-transfected with miR-12 and pIZ/EGFP-PTEN-3′UTR remarkably decreased compared with the controls (Figure 3B), showing that miR-12 directly interacted with PTEN. To evaluate the interaction between miR-12 and *PTEN in vivo*, miR-12 was overexpressed or knocked down, followed by the assessment of *PTEN* mRNA and protein levels. The data revealed that miR-12 overexpression led to a significant reduction in *PTEN* transcript and protein levels, while miR-12 silencing significantly increased the PTEN mRNA and protein levels (Figure 3C). These results showed that miR-12 could target *PTEN*.

To characterize the role of PTEN in phagocytosis, the expression of PTEN was knocked down, followed by an assessment of the antiviral phagocytic activity of shrimp hemocytes. The results showed that the injection of PTEN-siRNA resulted in a significant reduction of the *PTEN* transcript level (Figure 3D), indicating that *PTEN* expression was silenced by PTEN-siRNA. Western blots yielded similar results (Figure 3D). When PTEN was silenced, the shrimp phagocytic activity against WSSV was dramatically strengthened compared with controls (Figure 3E), indicating that PTEN was required in shrimp phagocytosis. Further results showed that PTEN silencing led to the remarkable suppression of WSSV replication in contrast with the controls (Figure 3F). These data revealed that PTEN exerted negative effects on the antiviral phagocytosis of shrimp.

The above findings showed that miR-12 could enhance the antiviral phagocytosis of shrimp by directly targeting *PTEN*, a phagocytosis negative regulation gene.

### The Enhancement of the Antiviral Apoptotic Activity of Shrimp Mediated by miR-12 through Targeting the Shrimp *BI-1* Gene

**Figure 4:**
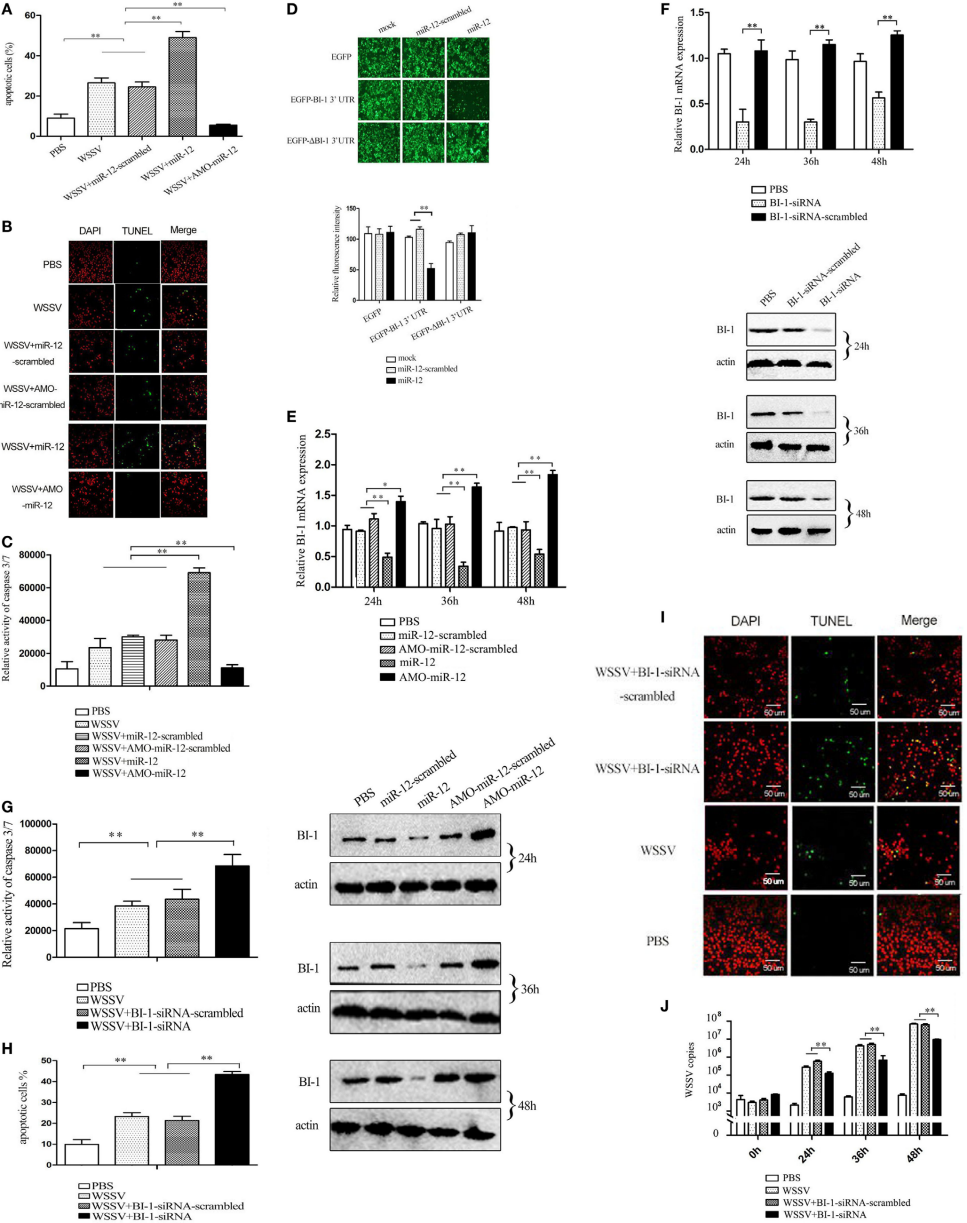
The enhancement of the antiviral apoptotic activity of shrimp mediated by miR-12 through targeting the shrimp *BI-1* gene. **(A)** The effects of miR-12 on apoptosis. Shrimp were injected with miR-12 + white spot syndrome virus (WSSV) or AMO-miR-12 + WSSV. At 36 h after injection, the shrimp hemocytes were subjected to annexin V analysis. **(B)** The evaluation of the apoptotic activity of shrimp hemocytes using TUNEL assay. **(C)** The detection of caspase 3/7 activity in shrimp hemocytes. **(D)** The direct interaction between miR-12 and BI-1 *in vitro*. Insect High Five cells were simultaneously transfected with miR-12 and different recombinant plasmids. At 48 h after transfection, the fluorescence intensities of the cells were detected. **(E)** The interaction between miR-12 and BI-1 *in vivo*. Shrimp were injected with synthesized miR-12 or AMO-miR-12 to overexpress or knockdown the expression of miR-12. At different times after injection, the BI-1 expression level was assessed using quantitative real-time PCR (up) and western blotting (down). In the western blots, the numbers indicate the times postinfection. Shrimp β-actin was used as a control. **(F)** The silencing of BI-1 expression in shrimp. Shrimp were injected with BI-1-siRNA. BI-1-siRNA-scrambled was used as a control. At different times after injection, the expression level of BI-1 was examined using quantitative real-time PCR (up) and western blotting (down). **(G)** The influence of BI-1 silencing on the caspase 3/7 activity in shrimp. Shrimp were co-injected with BI-1-siRNA and WSSV. At 36 h after injection, the caspase 3/7 activity in shrimp hemocytes was evaluated. **(H)** The detection of apoptosis of BI-1-silenced shrimp hemocytes with annexin V. **(I)** The evaluation of the apoptosis of BI-1-silenced shrimp hemocytes using TUNEL. Scale bar, 50 µm. **(J)** The impact of BI-1 silencing on virus infection. The expression of BI-1 in WSSV-infected shrimp was knocked down using BI-1-siRNA. At different times postinfection, the WSSV copy number in shrimp hemocytes was evaluated using quantitative real-time PCR. In all panels, the data are representative of three independent experiments (*p < 0.05; **p < 0.01).

To evaluate the effects of miR-12 on apoptosis, miR-12 was overexpressed or knocked down in shrimp, and then the apoptotic activity of shrimp hemocytes was assessed. The results of annexin V analysis showed more annexin V-positive hemocytes in WSSV-infected shrimp than in PBS-treated shrimp (Figure 4A). The miR-12 overexpression augmented the percentage of apoptotic hemocytes in contrast with that of shrimp injected with miR-12-scrambled and WSSV or WSSV alone (Figure 4A), suggesting that miR-12 played a positive role in host cell apoptosis. Contrarily, miR-12 silencing diminished the abundance of the annexin V-positive hemocytes (Figure 4A). TUNEL assays and the detection of caspase 3/7 of shrimp hemocytes yielded results similar to those obtained in the annexin V analysis (Figures 4B,C). These results showed that miR-12 was involved in the positive regulation of apoptosis in shrimp.

As predicted, the *PARP1* and *BI-1* genes encoding inhibitors of apoptosis were potential targets of miR-12 (Figure S2A in Supplementary Material). To explore the underlying mechanism of miR-12-mediated antiviral apoptosis, the interactions between miR-12 and *PARP1* and *BI-1* were characterized.

Recombinant pIZ/EGFP-BI-1-3′UTR and pIZ/EGFP-ΔBI-1-3′UTR were constructed (Figure S3 in Supplementary Material), and the recombinant plasmids were co-transfected with miR-12 or miR-12-scrambled into insect cells. The results indicated that the fluorescence intensity of the cells co-transfected with pIZ/EGFP-BI-1-3′UTR and miR-12 was significantly decreased compared with the controls (Figure 4D), showing that miR-12 directly interacted with BI-1. To determine whether miR-12 interacted with *BI-1 in vivo*, miR-12 was overexpressed or silenced in shrimp, followed by an assessment of the BI-1 expression level. The results showed that miR-12 overexpression significantly reduced BI-1 expression levels compared with the controls, while knocking down miR-12 expression by injection with AMO-miR-12 significantly upregulated BI-1 expression in shrimp (Figure 4E). These data indicated that miR-12 interacted with BI-1 *in vivo*.

To investigate the interaction between PARP1 and miR-12, the synthesized miR-12 and the pIZ/EGFP-PARP1-3′UTR construct were co-transfected into insect cells. The results showed no significant difference in the fluorescence intensity between various treatments (Figure S4 in Supplementary Material). The *in vivo* assays revealed that the PARP1 expression level did not change in shrimp when miR-12 was overexpressed or silenced (Figure S4 in Supplementary Material). These results indicated that miR-12 did not interact with PARP1. Therefore, miR-12 could promote apoptosis by targeting the *BI-1* gene.

To further reveal the role of BI-1 in apoptosis, BI-1 expression was knocked down by BI-1-siRNA, followed by evaluation of apoptosis. The results showed that the BI-1 expression in shrimp was silenced using BI-1-siRNA (Figure 4F), indicating the specificity of BI-1-siRNA. BI-1 silencing significantly increased caspase3/7 activity in shrimp hemocytes compared with the controls (Figure 4G), showing that BI-1 exerted an inhibitory effect on apoptosis. The data from the annexin V and TUNEL assays yielded results similar to that of caspase 3/7 activity detection (Figures 4H,I). These findings revealed that BI-1 was an inhibitor of apoptosis.

To explore the role of BI-1 in virus infection, the expression of BI-1 was silenced by BI-1-siRNA, and then the WSSV copy number in shrimp was assessed. The results revealed that BI-1 silencing significantly suppressed the proliferation of WSSV compared with the controls (Figure 4J). These data indicated that BI-1 exerted a positive effect on WSSV infection in shrimp.

The above findings suggested that miR-12 could trigger the antiviral apoptosis of shrimp by downregulating expression of the *BI-1* gene, which encodes an inhibitor of apoptosis.

### The Mechanism Underlying the miR-12-Mediated Synchronous Triggering of Multiple Antiviral Pathways

**Figure 5:**
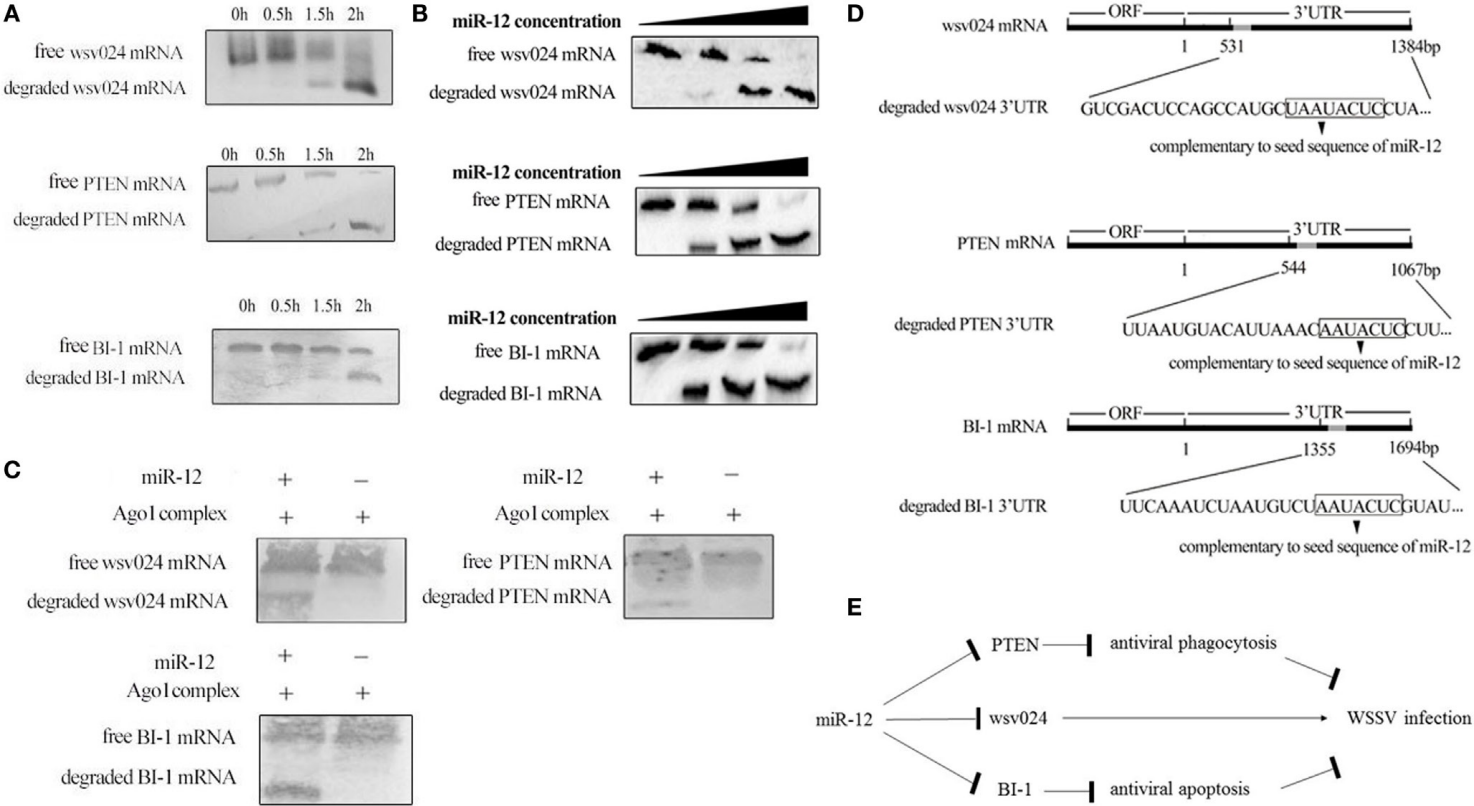
The underlying mechanism of miR-12 synchronously triggering multiple antiviral pathways. **(A)** Time-course degradation of miR-12 target mRNAs in the shrimp Ago1 complex. The 3′ untranslated region (3′UTR) of *wsv024, PTEN*, or *BI-1* mRNA, miR-12, and the Ago1 complex were incubated for various times (0, 0.5, 1, and 2 h). Subsequently, *wsv024, PTEN*, and *BI-1* mRNAs were detected using northern blotting. **(B)** The effects of the miR-12 concentration on the degradation of target mRNAs. The 3′UTR of *wsv024, PTEN*, or *BI-1* mRNA and the Ago1 complex were incubated with miR-12 at different concentrations for 2 h, followed by Northern blot analysis to detect the mRNAs. **(C)** The miR-12-mediated synchronous degradation of target mRNAs. The 3′UTRs of *wsv024, PTEN*, and *BI-1* mRNAs were mixed in equal amounts, followed by incubation with miR-12 and the Ago1 complex for 2 h. The mRNA degradation was monitored using northern blotting. **(D)** The sequencing of degraded target mRNA (*wsv024, PTEN*, and *BI-1*) 3′UTRs mediated by miR-12. **(E)** Proposed model for the host miR-12-mediated triggering of multiple antiviral pathways.

The above data showed that shrimp miR-12 promoted antiviral phagocytosis and antiviral apoptosis and inhibited virus infection by targeting the shrimp genes *PTEN* and *BI-1* and the viral gene *wsv024* in shrimp. To further explore the mechanism of the miR-12-mediated simultaneous triggering of multiple antiviral pathways in shrimp, the miR-12-mediated degradation of three different target genes was evaluated. The results of time-course experiments showed that the amount of degraded fragments of three target mRNAs increased with extended reaction time (Figure 5A), indicating that miR-12 mediated the degradation of its target mRNAs in the Ago1 complex. Additional data revealed that miR-12 mediated the degradation of three target mRNAs in a miRNA-concentration-dependent manner (Figure 5B).

To evaluate whether miR-12 mediated the synchronous degradation of multiple target mRNAs, the 3′UTRs of *PTEN, BI-1*, and *wsv024* mRNAs were mixed at equivalent amounts and then incubated with miR-12 and the Ago1 complex. Northern blots indicated that the three mRNAs were simultaneously degraded (Figure 5C). These data showed that miR-12 could mediate the synchronous degradation of multiple target genes. To confirm the miR-12-mediated degradation of target mRNAs, the degraded 3′UTR fragments of *wsv024, PTEN*, and *BI-1* mRNAs were sequenced. The results indicated that miR-12 could mediate the 5′–3′ exonucleolytic degradation of its target mRNAs, and the miR-12-mediated degradation terminated at a vicinity of the 3′UTR sequence complementary to the seed sequence of miR-12 (Figure 5D).

Taken together, the findings showed that miR-12 simultaneously triggered phagocytosis, apoptosis, and antiviral immunity through synchronous targeting of shrimp genes (*PTEN* and *BI-1*) and a viral gene (*wsv024*) (Figure 5E).

## Discussion

Virus infection is the major origin of many diseases. To protect the host from virus invasion, the host gradually evolves a series of antiviral mechanisms in response to virus attack. When a virus invades its host or has been initiated to replicate in target cells, multiple antiviral immune pathways of the host are triggered to ensure the clearance of pathogens (24, 25). Owing to the diversity of antiviral immune mechanisms, various trigger factors may be released to initiate multiple antiviral pathways in the host. Toll-like receptors 3, 7, and 8 accumulate when pathogens invade, thus promoting the host antiviral responses through the induction of specific genes, such as interferon regulatory factors (IRFs) and nuclear factor of kappa light polypeptide gene enhancer in B cells (NF-κB) (24). Double-stranded RNA (dsRNA) represents a molecular intermediate during virus replication within infected cells (25). The existence of dsRNA in the cytoplasm can induce the release of RNA helicases, such as RIG-I (DExD/H-box helicase 58), which are used to detect cytoplasmic dsRNA and trigger the production of antiviral molecules, including type I interferons. However, whether an individual molecule can synchronously trigger different host antiviral pathways remains unclear. In the present study, the results indicated that shrimp miR-12 triggered shrimp antiviral apoptosis and phagocytosis through interactions with host *BI-1* and *PTEN* genes and simultaneously inhibited WSSV replication by targeting the viral *wsv024* gene. Therefore, the present study revealed that multiple host antiviral pathways could share the same trigger factor (i.e., miRNA). Due to its multiple target genes, a single miRNA revealed the synchronous regulation mechanism of multiple antiviral immune pathways.

The results of the present study revealed that shrimp miR-12 synchronously initiated multiple antiviral pathways by directly targeting the virus *wsv024* gene and the host *PTEN* and *BI-1* genes. During phagocytosis, PTEN abrogates ITAM (immunoreceptor tyrosine-based activation motif)-dependent signaling events *in vivo* and controls RAC activities to inhibit phagocytic capacities, showing negative regulation on antiviral phagocytosis (26). In fungi, PTEN directly activates the actin depolymerization factor CFL1 (cofilin-1) during PGE2-mediated inhibition of phagocytosis (23). BI-1, an inhibitor of the apoptotic activator BAX (BCL2 associated X), can suppress apoptosis (27). In the present study, the results indicated that the viral *wsv024* gene was required for WSSV infection. Therefore, the miR-12 target genes *wsv024, PTEN*, and *BI-1* played negative roles in the antiviral immunity of shrimp. The results of the present study demonstrated that miR-12 mediated the synchronous degradation of three target genes (*wsv024, PTEN*, and *BI-1*) in the shrimp Ago1 complex, demonstrating the simultaneous regulation of multiple antiviral immune pathways. As previously reported, miRNAs mediate target gene silencing through the induction of mRNA degradation or transcriptional repression, and target mRNA degradation affords a dominating contribution to silencing (28–30). In plants, miRNAs directly induce the endonucleolytic cleavage of full complementary mRNAs in the middle of the matching regions of their target genes (31). However, in animals, the majority of target mRNAs are incompletely complementary. In animals, miRNAs can guide their targets to the cellular 5′- to 3′-exonucleolytic digestion of mRNAs (32–34), consistent with the findings of the present study. In the case of 5′- to 3′-exonucleolytic degradation, mRNAs are initially deadenylated by the CAF1 (CCR4-NOT transcription complex subunit 8)-CCR4 (C–C motif chemokine receptor 4)-NOT deadenylase complex, followed by decapping *via* the enzyme DCP2 (decapping mRNA 2) (35). DCP2 typically exerts its decapping function with the aid of other co-factors, such as DCP1 (decapping mRNA 1), EDC4 (enhancer of mRNA decapping 4), and the DEAD-box protein RCK (also known as Me31B), for full activity or stability. However, the mechanism of the miRNA-mediated deadenylation of mRNAs remains unclear. In this context, the present study contributed novel insights to the miRNA-mediated simultaneous regulation of multiple antiviral pathways in invertebrates.

## Author Contributions

XZ conceived the project. LS performed the experiments and wrote the paper.

## Conflict of Interest Statement

The authors declare that the research was conducted in the absence of any commercial or financial relationships that could be construed as a potential conflict of interest. The reviewer, AT, and handling editor declared their shared affiliation, and the handling editor states that the process nevertheless met the standards of a fair and objective review.
