# Supplementary material for: Shrimp miR-12 Suppresses White Spot Syndrome Virus Infection by Synchronously Triggering Antiviral Phagocytosis and Apoptosis Pathways
Source: Front Immunol. 2017 Jul 31;8:855. doi: 10.3389/fimmu.2017.00855 (PMC5534442; doi:10.3389/fimmu.2017.00855)
Supplement: Supplementary file 1 [file Presentation_1.PDF]

Supplementary figures

Fig S1. The prediction of WSSV genes targeted by miR-12 and constructs of target gene 3'UTRs. (A) The prediction of the WSSV genes targeted by miR-12. The results indicated that the 3'UTR of the *wsv024* gene could be targeted by miR-12. **(B)** Construction of recombinant plasmids containing EGFP and the 3'UTR of wsv024. The sequence complementary to the seed region of miR-12 is underlined.

Fig S2. The prediction of shrimp genes targeted by miR-12 and constructs of target gene 3'UTRs. (A) The prediction of shrimp genes targeted by miR-12. As predicted, the 3'UTRs of *PARP1*, *PTEN* and *BI-1* genes could be targeted by miR-12. **(B)** Constructs of pIZ/EGFP-PTEN 3'UTR and pIZ/EGFP-ΔPTEN 3'UTR. The underlining indicates that the 3'UTR region of PTEN matched the seed sequence of miR-12.

Fig S3. Sketch map of recombinant plasmids. The sequence of BI-1 3'UTR complementary to the seed region of miR-12 is underlined.

Fig S4. The interaction between miR-12 and PARP1. Insect High Five cells were simultaneously transfected with miR-12 and the construct EGFP-PARP1 3' UTR. At 48 h post-transfection, the fluorescence intensities of transfected cells were examined (up). To explore the interaction between miR-12 and PARP1 *in vivo*, shrimp were injected with miR-12 or AMO-miR-12. At different times after injection, the BI-1 expression level was assessed using quantitative real-time PCR (down).

Fig S1

A

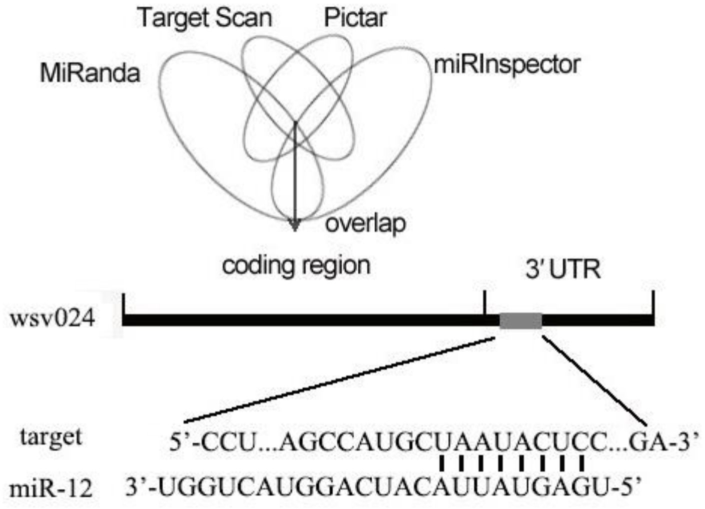
Target Scan
Pictar
MiRanda
miRInspector
overlap
coding region
3′UTR
wsv024
target
5'-CCU...AGCCAUGCUAAUACUCC...GA-3'
miR-12
3'-UGGUCAUGGACUACAUUAUGAGU-5'

B

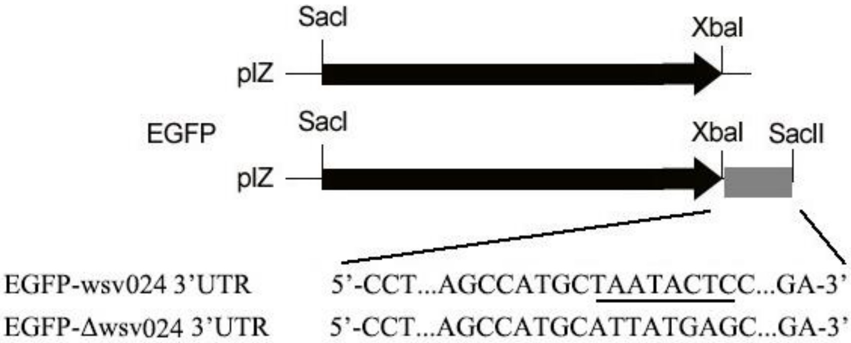
SacI
XbaI
pIZ
EGFP
SacI
XbaI
SacII
pIZ
EGFP-wsv024 3'UTR
5'-CCT...AGCCATGCTAATACTCC...GA-3'
EGFP-Δwsv024 3'UTR
5'-CCT...AGCCATGCATTATGAGC...GA-3'

Fig S2

A

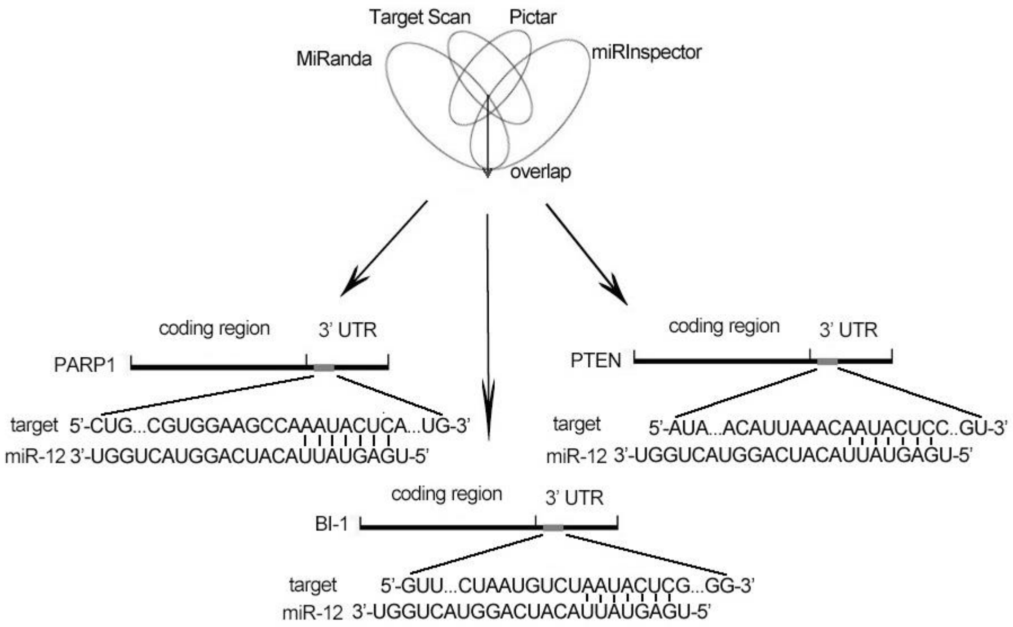
Target Scan
Pictar
MiRanda
miRInspector
overlap
coding region
3' UTR
PARP1
target 5'-CUG...CGUGGAAGCCAAAUACUCA...UG-3'
miR-12 3'-UGGUCAUGGACUACAUUAUGAGU-5'
coding region
3' UTR
PTEN
target 5'-AUA...ACAUUAAACAAUACUCC..GU-3'
miR-12 3'-UGGUCAUGGACUACAUUAUGAGU-5'
coding region
3' UTR
BI-1
target 5'-GUU...CUAAUGUCUAAUACUCG...GG-3'
miR-12 3'-UGGUCAUGGACUACAUUAUGAGU-5'

B

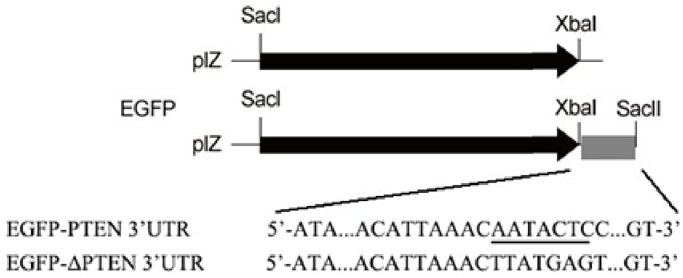
SacI
XbaI
pIZ
EGFP
SacI
XbaI
SacII
pIZ
EGFP-PTEN 3'UTR 5'-ATA...ACATTAAACAATACTCC...GT-3'
EGFP-ΔPTEN 3'UTR 5'-ATA...ACATTAAACTTATGAGT...GT-3'

Fig S3

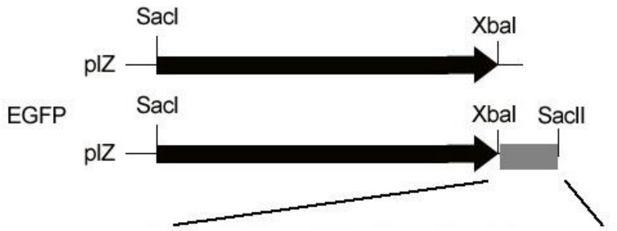

EGFP-BI-1 3'UTR 5'-GTT...GTAATGTCTAATACTCG...GG-3'
EGFP-ΔBI-1 3'UTR 5'-GTT...CTAATGTCTTTATGAGG...GG-3'

Fig S4

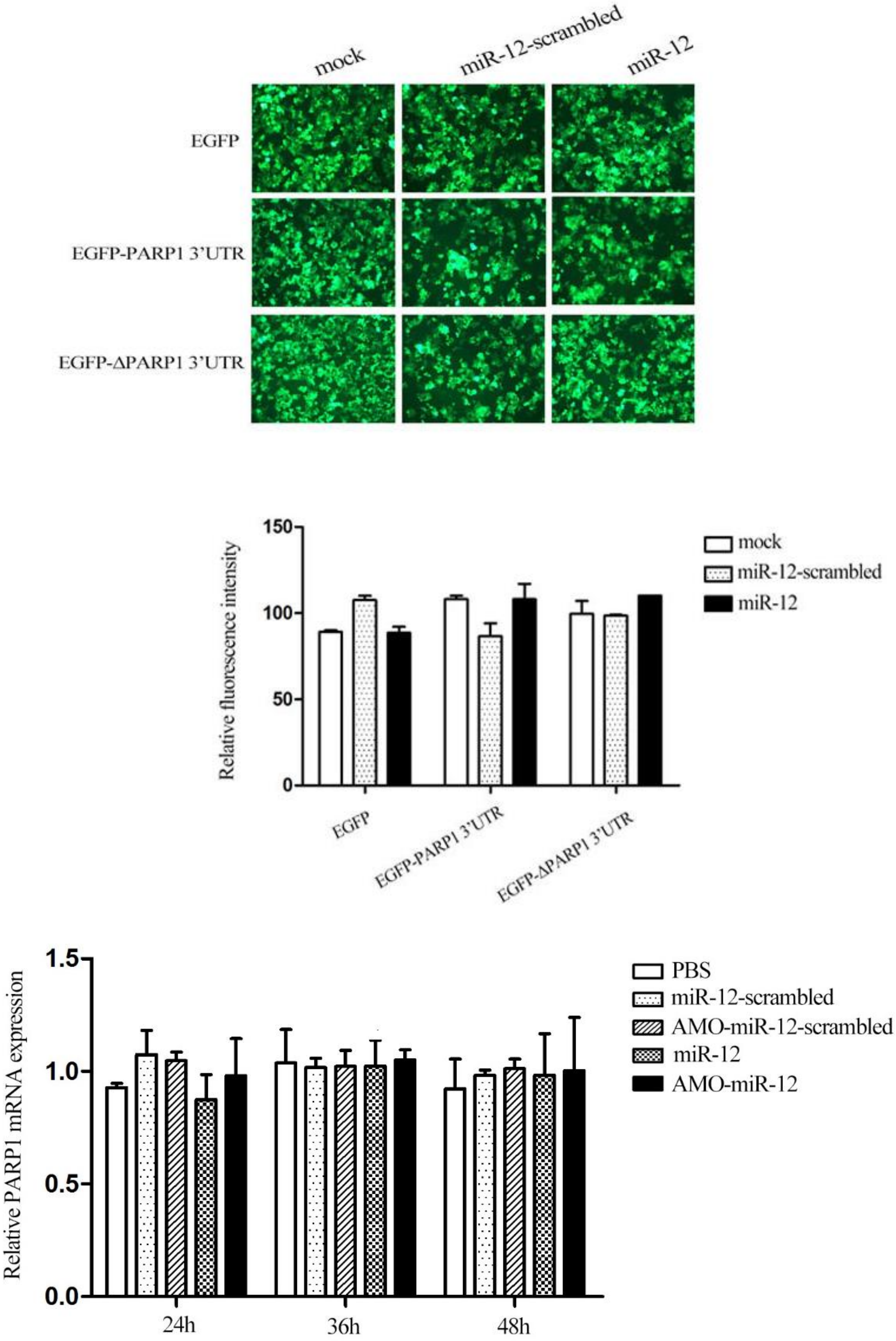

mock
miR-12-scrambled
miR-12
EGFP
EGFP-PARP1 3'UTR
EGFP-ΔPARP1 3'UTR
Relative fluorescence intensity
150
100
50
0
mock
miR-12-scrambled
miR-12
EGFP
EGFP-PARP1 3'UTR
EGFP-ΔPARP1 3'UTR
Relative PARP1 mRNA expression
1.5
1.0
0.5
0.0
24h
36h
48h
PBS
miR-12-scrambled
AMO-miR-12-scrambled
miR-12
AMO-miR-12
